# Peer Support Workers in Mental Health Care: Plural Positionings Beyond Transformation and Assimilation Dichotomy

**DOI:** 10.1111/hex.70515

**Published:** 2026-01-15

**Authors:** Ludovica Aquili, Michele Rocelli, Susanna Brunelli, Federica Mangione, Milena Negri, Giuseppe Salamina, Elena Faccio

**Affiliations:** ^1^ Department of Philosophy, Sociology, Education and Applied Psychology University of Padua Padua Italy; ^2^ Mad in Italy Diritti alla follia APS Italy; ^3^ Associazione Italiana Persone Esperte in Supporto tra Pari (AIPESP); ^4^ Associazione Italiana Persone Esperte in Supporto tra Pari (AIPESP); Associazione Sentiero Facile Reggio Emilia Reggio Emilia Italy; ^5^ Department of Mental Health Local Health Unit of Turin Turin Italy

**Keywords:** collaborative research, co‐writing, identity, lived experience, peer support workers, positioning theory, recovery

## Abstract

**Introduction:**

The role of the Peer Support Worker (PSW) has gained increasing prominence in mental health services, yet its institutionalisation raises questions about balancing autonomy and assimilation into service logics. This study examines the narrative positionings of three PSWs, focusing on how they construct professional identity in relation to mandate, collaboration with practitioners, and service organisation.

**Methods:**

A qualitative design was adopted using semi‐structured interviews with three PSWs. Data were analysed through Positioning Theory (Davies and Harré 1999) and Bamberg's three‐level model (2022), which investigates positioning in relation to temporality, the other and agency. Particular attention was given to positions regarding the professional mandate, collaboration with practitioners, psychiatric and psychotherapeutic practices, and the services in which participants had placements or are currently engaged.

**Results:**

PSW positioning emerged as dynamic and multifaceted, constantly negotiated between institutional mandates and fidelity to lived experience. Three configurations were identified: (1) a critical and independent stance, maintaining symbolic distance from services whilst collaborating with them; (2) an integrated stance, balancing institutional logics with attentiveness to user experience; and (3) a fluid stance, affirming the coexistence of PSW and service‐user roles and challenging conventional boundaries between clinical and experiential knowledge. Across all narratives, PSWs located themselves in liminal spaces that foster transformative possibilities for mental health services.

**Conclusion:**

PSW identity is not fixed but articulated through multiple positionings, ranging from critical autonomy to institutional integration and fluid coexistence of service‐user and professional roles. This plurality, rather than constituting ambiguity, represents an epistemic and ethical resource that extends the transformative potential of services beyond the dichotomy of transformation versus assimilation. Recognising this plurality means valuing PSW agency and fostering flexible, participatory practices responsive to the complexity of lived experience.

**Involving the Participants:**

The research team included both academics and PSWs in a co‐participatory design initiated through voluntary applications. Only interviews with explicit consent were included. The first draft of the analyses was submitted for participant validation: each PSW reviewed their own transcript and related comments, with the option to modify or expand the analysis. Materials were then shared within the group to enable collective reflection and refinement of interpretation. Through this collective process, the group co‐authored the present version of the article.

**Clinical Trial Registration:**

Does not fit.

## 1 Introduction

Over the past two decades, experiential knowledge has gained increasing recognition as an embodied and legitimate form of knowledge in mental health contexts, capable of complementing—and at times challenging—traditional clinical epistemologies [1, 2]. Within this landscape, the innovative role of the Peer Support Worker (PSW) has emerged, integrating direct experience of mental distress into helping relationships grounded in recovery, reciprocity, empowerment and lived testimony [3].

The inclusion of PSWs in psychiatric services follows heterogeneous models, influenced both by international guidelines [4] and by the specificities of local contexts (Faccio et al. 2024). The professional identity of PSWs thus emerges as the outcome of a dynamic negotiation between individual subjectivity and organisational norms, through a continuous process of discursive, ethical and professional positioning [5, 6, 7].

International literature has highlighted the ambivalent nature of PSW integration, marked by a constant tension between the drive for participatory innovation and the tendency towards institutional assimilation. The process of institutionalisation—aimed at legitimising the role through professional standards, accredited training, supervision systems and contractual recognition—responds to the need to ensure stable and recognised integration within services ([8]; Jones et al. 2021). However, this dynamic may conflict with the relational, horizontal and mutualistic nature of peer support, generating tensions with the bureaucratic and hierarchical logics of clinical contexts [9, 10]. Some studies have further shown that a professionalisation process lacking critical reflection risks undermining the authenticity of the role and compromising its transformative potential [11].

Research consistently shows that, once embedded in services, PSWs are often channelled into operational tasks governed by prescriptive protocols and performance metrics that limit autonomy and reduce reciprocity to mere service provision [12, 13, 14, 15, 16]. At the same time, neoliberal governance devices and standardisation processes encourage their co‐optation into clinical bureaucracies, reducing their ability to challenge dominant forms of knowledge and weakening the transformative potential of the role [17, 18, 19, 20]. Current neoliberal dynamics permeating services profoundly shape this process. Baines and Van Den Broek [21] highlight how PSWs may be integrated in ways functional to the system, reinforcing—rather than questioning—existing practices. In this context, experiential knowledge risks being treated as a new form of expertise to be normalised, whilst standardisation may reduce the role to the execution of predefined protocols [22], fuelling concerns over progressive homogenisation into the traditional professional model ([3]; Davidson et al. 2012; [23]).

The recovery paradigm has profoundly transformed the conception of care in mental health services, shifting the focus from symptom remission to valorising lived experience and constructing personal meaning‐making journeys centred on the service user's voice (Anthony 1993; Davidson et al. 2006; [24]). Recovery‐oriented practices emphasise supporting people in living purposeful and meaningful lives despite the presence of mental distress or persistent symptoms (Leamy et al. 2011). Within this framework, the role of the PSW has been established. However, various studies have shown that once institutionalised, the recovery paradigm risks being absorbed into the organisational and governance logics of services, losing part of its original transformative value: once incorporated into organisational routines, it can act as a form of biopolitical regulation that restricts acceptable identities and practices (Rose 2014; [25]; Piat and Lal 2012). This dynamic is also reflected in the autobiographical narratives of PSWs, often shaped by institutional cultural codes [26, 27]. In other words, the focus shifts from individual performances to the organisational frameworks that make a plural and user preference‐driven recovery practicable.

Whilst institutional recognition fosters legitimacy and inclusion, increasing professionalisation may also undermine experiential distinctiveness, generating identity conflicts between the role of peer and that of professional [28, 29, 30]. Within this framework, one can speak of a true identity transformation, in which the adoption of organisational languages and behaviours by PSWs occurs at the cost of a progressive erosion of their critical voice [31].

The risk is not only systemic absorption and the loss of transformative capacity, but also the reproduction of pre‐existing logics rather than their disruption [8, 32, 33]. For this reason, numerous studies emphasise the importance of accompanying integration and professionalisation processes with a critical posture, one capable of preserving the radicality of experiential knowledge [11, 12].

This contribution situates itself within this debate by exploring the perspectives of three PSWs trained in a structured programme for PSWs. This represents the first national‐level training implemented in Italy (Borgonuovo, Bologna, 2023–2024), funded by the Ministry of Health, in contrast to previous initiatives developed primarily at local or regional levels. It is important to note that, in the Italian context, the integration of PSWs within mental health services remains marginal. Only a limited number of services have begun implementing WHO recommendations by promoting their inclusion. Thus, Italy is still in the early stages of recognising the PSW role, with experiences restricted to local initiatives [34]. In this context, at the time of data collection, there was no ministerial regulation or guideline governing whether a PSW could work in the same service where they are or have been treated. As this is an emerging field, the orientation is delegated to individual services, which define local practices on a case‐by‐case basis.

Training offers a privileged lens for examining the tensions surrounding this role: it not only conveys competencies but also profoundly shapes the perceived mandate and vision of the role. Understanding how training influences the construction of PSWs' professional subjectivity is crucial to grasping their positioning within services and the transformative potential of shared experiential knowledge.

This study reconstructs the process through which the participants became PSWs. The analysis focuses on the positions assumed during three temporal periods—Entry into the training programme, Training experience and Enactment of the PSW role in service contexts—in relation to the professional mandate, collaboration with professionals, psychiatric and psychotherapeutic practices, and the services in which they completed their internship or are currently employed.

### 1.1 Theoretical Background

The construct of positioning is here understood in line with the perspective of Davies and Harré, who define it as ‘*the discursive process whereby selves are located in conversations as observably and subjectively coherent participants in jointly produced storylines*’ (Davies and Harré, 1999, p. 37). Positioning thus represents a dynamic mode of identity construction, situated within narrative interaction.

Within this framework, Bamberg's approach (2022) distinguishes three levels of positioning through which the sense of self is constructed in everyday practices:

1. Temporality, namely how subjects situate themselves in time, emphasising transformations, ruptures or continuities;
2. The relationship with the other, whereby the narrated self is defined through similarity, distance or opposition;
3. Agency, that is, the tension between being determined by external circumstances and presenting oneself as a conscious and agentive actor in one's own trajectory.

In our view, Positioning Theory proves particularly effective in this context as it avoids reifying the role of PSWs and provides an interpretative lens through which to grasp its complexity, dynamic character and still‐evolving definition. As mentioned above, in the Italian context, the PSW role is in the process of formation and is subject to significant variation in the ways it is structured.

The National Charter of PSWs, drafted during the First National Conference of Users and Families (2021), marked an initial step towards formal recognition, involving 43 associations across 11 regions. A further advancement took place in 2023, when the Ministry of Health promoted the first national‐level training initiative. Despite these developments, the process of institutionalisation remains embryonic. Given the heterogeneity of practices and interpretations that shape the role, examining the different narrative positionings allows for an exploration of such variations whilst preserving and valuing their plurality.

### 1.2 Study Aim

This study offers an original and systematic perspective on the narrative positionings of three PSWs, focusing on their training pathways and direct experiences in the role. Building on insights from the literature, the aim is to explore how PSWs position themselves in relation to the professional mandate, collaboration with other service practitioners, the modalities through which the role is enacted, and the broader mental health services. Specifically, the study investigates their narrative positioning in relation to two key polarities. On the one hand, it examines the assimilation of an already existing professional model, with a mandate oriented towards entering traditional services to promote change from within, through practices and relationships. On the other hand, it explores the creation of an innovative and autonomous role, aligned with a more radical theoretical mandate, aimed at redefining the very epistemological and operational foundations of the services themselves.

## 2 Materials and Methods

### 2.1 Context and Participants

This article is part of a broader project that initially involved 22 participants in the Italian training programme for PSWs, conducted between 2022 and 2023 and based on the German Ex‐In (Experienced Involvement) model (https://ex-in.de/). The project pursued both evaluative and exploratory aims (publication in progress). Over time, several parallel initiatives were launched: some to support recognition of the PSW role through AIPESP—Italian Association of Experts in Peer Support (established 6 June 2025); others to develop training proposals in mental health services; and still others, as in this case, to foster collaborations with PSWs in research.

The three participants in this study, all Ex‐In graduates, had prior experience in peer support, which the training further enhanced by highlighting differentiated modalities of role enactment. They showed a strong interest in taking an active part in the above projects. Their engagement thus reflects a pre‐existing commitment to collective reflection and critical reconsideration of PSW inclusion, while offering an opportunity to explore diverse interpretations and practices of the role. Their participation enabled a co‐constructed reading of the data, enriching the depth of analysis. Table 1 summarises the demographic and professional characteristics of the participants.

**Table 1:** Personal and professional characteristics of the participants.

Participant	Age	Gender	Years of work as PSW	Current occupational status	Work setting
S.	62	F	6 years as PSW	Volunteer/activist	Advocacy: works independently, providing individual support through listening and accompaniment pathways.
M.	60	F	3 years as PSW	Internship contract at the mental health service	Clinical setting: coordination of the day centre, telephone reception at the mental health centre, work in the psychiatric ward, and employed by services for home‐based user support.
F.	38	F	6 years as PSW	Internship contract within a work inclusion project at an association	Clinical–social setting: PSW in recreational workshops within an association, and PSW collaborating with the Department of Mental Health.

### 2.2 Data Collection

This qualitative study is based on interviews conducted in February 2024 with three PSWs. Semi‐structured interviews were carried out in person. To explore how participants construct their role as PSWs, the interviews addressed the following areas:

1. Entry into the training programme: participants were asked to recount their entry into the training course, to understand how this moment intersected with previous experiences of mental distress, their pathways through services as users, and their relationships with service staff, as well as to elicit expectations and motivations for undertaking the course.
2. Training experience: we explored how participants experienced the training in light of their personal vision of the PSW role and how the training contributed to redefining their conception of the role.
3. Enactment of the PSW role in service contexts: the aim was to understand the experiences that illustrate the specific ways in which each participant enacted the PSW role across different operational contexts.

Table 2 shows the interview questions for each research area. The guide served only as a flexible framework, with wording adapted to each participant.

**Table 2:** Research areas, analytical objectives and guiding interview questions.

Research area	Analytical object	Guiding questions
1. Entry into the training course	To explore how participants narrate their entry into the training course, to understand how this moment intersects with previous experiences of mental distress, service use as a patient and relationships with service staff, as well as to elicit expectations and motivations for undertaking the course.	‒ *Could you tell me how you came to know about this course, and what led you to start it?* ‒ *What thoughts or feelings did you have when you decided to enrol?* ‒ *In what ways did your personal history influence your decision to participate?*
2. Training experience	To investigate how participants experienced the training and how it influenced their vision of the PSW role.	‒ *How would you describe your training experience?* ‒ *What were the most relevant moments for your trajectory as a PSW?* ‒ *In what ways did the training help you to ‘be a PSW’ in your daily work?*
3. Enactment of the PSW role in different contexts of intervention	To analyse how participants interpret and enact the PSW role in operational contexts (e.g., services, teams and relationships with users).	‒ *How are you perceived and welcomed in the contexts where you work or collaborate?* ‒ *Are there situations in which you feel ‘at ease’ or ‘out of place’ in the role?* ‒ *What helps or hinders you in putting into practice what you learned?* ‒ *Could you describe a significant episode in which you truly felt like a PSW?*

### 2.3 Data Analysis

The textual material was analysed using the analytical framework of positioning [35, 36]. Each of the three research areas was examined through narrative excerpts from the interviews, treated as ‘small stories’ [37], defined as ‘*situated language use, employed by speakers to organize a visualization of situated and context‐bound identities’* (p. 2). This approach enabled us to investigate the processes of positioning and identity construction that emerged during the interaction [37]. Table 3 outlines the analytical process in detail.

**Table 3:** Analytical methodology: Levels of positioning analysis [36].

Level of positioning	Description of the analysis	Specific aspects observed
Positioning I—The story told	As indicated by the author, we first approached the textual material by observing the story being told; specifically, which characters were introduced and what kind of relationships they had with one another. At this level, particular emphasis was placed on how the main protagonist of the story—in this case, the interviewee in her biographical account—navigated the three identity spaces described above: *‘agency/passivity, sameness/difference, constancy/change’* (p. 9).	This level allowed us to observe how each participant positioned her PSW trajectory in continuity or in difference with respect to her personal history. We considered whether entry into the course was presented as an autonomous decision or as the result of recognition by others of her potential as a PSW (attributed positioning). We analysed the degree of agency, for example, through expressions of the desire to influence or change services, and how this was enacted: from within, through collaboration with services or from outside, as an *independent* subject. We also examined practices aimed at reshaping care relationships, contesting standardised procedures or taking a critical stance towards institutional asymmetry while asserting the transformative value of lived experience. Attention was paid to how participants positioned themselves in relation to other professional roles—as similar to or different from them—and to possible parallels or contrasts with service users. The linguistic dimension was central: word choices, pronouns and modes of expression were closely analysed. For example, a sense of self as a PSW can emerge when the role is defined in close collaboration with the service, as suggested by the use of the pronoun ‘*we’* (indicating ‘me and my service’).
Positioning II—Local and relational context	With regard to the second level—the description of the local and relational context of the interaction—since video recordings of the interviews were not available, we were able to focus exclusively on the linguistic means employed in the discursive performance.	This level of analysis allowed us to focus on the linguistic and performative means through which participants articulate their discursive performance and on the strategic use of the interview space, through which the parties negotiate their presence and legitimacy within the conversation. This approach enabled the identification of contextualisation cues (Gumperz 1982), that is, linguistic references useful for understanding how participants assume relational positions and orient the narrative.
		We observed that the use of direct discourse is not limited to reporting others' words or facts but becomes a resource for enacting social practices—such as denouncing problematic issues or negotiating recognition. In these passages, participants often stage other voices, against which they position themselves, for example, as competent and authoritative subjects. This construction of self as legitimate interlocutors also emerges through the use of emphatic locutions (‘I swear’) or assertive tones (‘I was already a PSW without knowing it’) with which they affirm discursive autonomy and experiential competence.
		These elements demonstrate how the dialogic exchange is not merely a transmission of content, but a performative act of identity, also facilitated by the relational context of the interview, in which the parties felt recognised and valued in their role as PSWs.
		We should specify that, since the interviews were designed to privilege the participants' narratives, the interviewer's intervention was limited to a few questions. As a result, we could not rely on frequent exchanges, as typically occurs in everyday conversations. It is also important to note that the use of semi‐structured interviews further contributed to limiting the scope of the exchanges.
Positioning III—Dominant discourses mobilised	After examining the participants' positionings in relation to both imagined and actual audiences, we then focused on the dominant discourses that were mobilised during the interactive exchange.	This level of analysis allowed us to investigate the role of dominant discourses in the construction of PSW identity. In particular, we observed whether, and which, discourses were mobilised to legitimise the role or, conversely, whether the self was presented as emancipated from internalised dominant discourses through emancipatory forms of positioning. For example, some subjectivities were defined through the dominant discourse on ‘*boundaries’* and ‘*distance’* to be maintained in professional contexts, as a strategy to safeguard one's role and regulate involvement with others. Other positionings asserted emancipation from the role of ‘*service user’,* explicitly contesting the idea that an ‘*user’* identity remains permanently anchored to the past crisis. Finally, some narratives articulated critical stances toward dominant psychiatric knowledge.

### 2.4 Ethical Considerations and Participant Involvement

Ethical considerations were a priority throughout the research process. The study received approval from the Ethics Committee for Psychological Research of the University of Padua (Area 17), protocol no. 4862. Participants provided written informed consent specifying the duration, objectives, procedures and audio‐recording of the interviews, in accordance with the requirements of Legislative Decree 196/2003 and the EU General Data Protection Regulation (GDPR) 2016/679.

Informed consent was collected at three stages: an initial stage, before the interviews and two subsequent stages, at the end of the analysis and the drafting of results. In these latter stages, each participant first reviewed the preliminary analysis individually and then collectively discussed their observations with the other two participants and the researchers. Through this collective process, the group collaboratively developed the results and discussion sections, culminating in the current version presented here. The three PSWs are co‐authors of this article.

This procedure, known as Validation and Participant Involvement (or member checking), is described in greater detail in Table 4.

**Table 4:** Validation and participant involvement.

Phase	Description	Specific actions undertaken
Phase 1—Individual validation (respondent validation)	To enhance the credibility and trustworthiness of the findings and to strengthen the ethical dimension of the research, we implemented respondent validation (Ritchie et al. 2013), namely the sharing and discussion of results with participants.	‒ Each participant received the analysis of her own interview (blinded with respect to the others). ‒ Individual consent was requested before group sharing. ‒ Participants indicated possible discrepancies or added elements. ‒ Comments were integrated:
		as direct modifications to the texts as visible notes.
Phase 2—Collective sharing	Once authorisation had been obtained, the narratives were made accessible to the entire group.	‒ The collective meeting offered a space for critical discussion. ‒ Additional interpretive issues emerged. ‒ The discussion contributed to the shared re‐elaboration of meanings. ‒ The reinterpretations were subject to collaborative writing [38, 39].

## 3 Results

The results are presented in three subsections, corresponding to key phases in the participants' journeys as PSWs: pre‐training, training and PSWs in action. For each phase, the narratives of S., M. and F. are analysed, highlighting how they position themselves and construct their PSW identity.

### 3.1 Pre‐Training

All participants positioned themselves as ready for the PSW role, but with distinct approaches: S. as a long‐standing advocate, M. seeking personal growth and validation, and F. as already embodying the role informally. Despite these differences, all reframed their lived experiences as valuable for the PSW role.

#### 3.1.1 S.'s Positioning: ‘Acceptance of the Letter as Proof of My Aptitude for Advocacy’

S., a woman in her early sixties, has encountered mental health services both as a user and as a family member, accumulating what she describes as ‘*25 years of experience’.* The following excerpt, considered as a *small story* (Georgakopoulou 2008), illustrates her reasons for undertaking PSW training:

I had 25 years of experience because I was also a family member, so I was always kind of fighting on these issues, you know!? But when I personally had to go through this myself—it lasted 18 months, but 18 months of hell, as I always say—it came naturally to me to transform this personal story into something that could be useful to someone else, because I've always had this inclination. So, when this friend of mine said: “look, there's this course,” I was like: “send me everything.”

[Regarding my application to the course, editorial note] ‘Anyway, I managed to get a letter of recommendation from the services, which I had … at the same time I was detaching from them, but I was still kind of there, so I asked them to write me this letter of recommendation. Then … I really prepared it carefully, I swear I worked on it really hard […] I must have spent more than 3 months preparing it […] It meant so much to me […]. It was a vision, my vision was: “I will go there.” I had already imagined myself being part of it, so much so that when I got this confirmation that I'd been accepted, I said: “wow, I did it.”’

Through this narration, S. positions herself in relation to her experience, transforming suffering from a private and clinical fact into a public and relational resource. Her personal and family history becomes the foundation of the PSW role, appearing as a choice already embedded in her biography before the training. Experiential identity emerges as inseparable from the role, legitimising suffering not as an obstacle but as competence.

Reflecting on the analysis, S. clarifies that her ambition was never traditional professional advancement, but a drive towards social and independent activism. Her candidacy thus becomes emblematic: the meticulous preparation of her application letter—over 3 months—illustrates a performative positioning that challenges the stereotype of the disorganised user. The time devoted marks a rupture from the identity imposed by diagnosis, positioning her as capable of agency, self‐determination and intentional planning. S. appropriates a space not institutionally foreseen, showing that access to the PSW role occurs not despite suffering but because of it, transforming vulnerability into competence and social commitment.

#### 3.1.2 M.'s Positioning: ‘The Beginning of the Course as Emancipation From Past Vulnerabilities’

M. first came into contact with mental health services in 2020. Following an initial experience of fragility, she began her pathway towards becoming a PSW:

My psychiatrist told me: “Look, there's this training course in Reggio Emilia (Northern Italy),” because he saw that I had developed an awareness of my illness that allowed me to start putting myself out there a little. I was very fearful and hesitant, because taking this course felt like going back to school. And I never finished school— in the third year of high school I dropped out because I wasn't well—so I didn't feel up to it. But then I said, come on, let's try, and I did it. […] Then they offered me the Ex‐In course. I talked about it with my tutors, I said yes, we want to do it, so we started preparing the letter of presentation, the application, and so on and so forth. The first reason was this: I wanted to do it for myself, to get to know myself better, to have more tools, to help myself in critical moments. When they accepted me, I thought: “Impossible. Impossible, they must have made a mistake.” I opened that email three or four times and … for me it was a beginning. Similar to the other course, but more motivated, because this one was really mine. Because I had chosen it, I had chosen to do it, and the people who had given me that brochure believed in me. And so, it became a very important journey for me.

Her narrative shows how M.'s initial positioning is largely shaped by external recognition received from the service, which identified her as ‘*aware’* and therefore suitable for the PSW role. The use of the first‐person plural signals how her new identity was constructed through institutional support, reflecting the expectations of the service itself.

The school metaphor guides M.'s narration, as she approaches the course as a space of identity transformation. Participation in EX‐IN training becomes, for her, a way to overcome the sense of inadequacy tied to her interrupted education, enabling her to reclaim competence and agency. The training thus represents a symbolic turning point, where initial fragility is transformed into confidence, social recognition and an awareness of her own capacity to carry through a project she had autonomously chosen.

#### 3.1.3 F.'s Positioning: ‘I Was Already a PSW Without Knowing It!’

As part of my rehabilitation project, I was included in a work placement program, a tailoring workshop run by an association. So, in reality, I was already working as a PSW without knowing it. I'm good with my hands, I was already helping others cut things properly—basically, I was being a PSW without knowing it, without any training. It all started very spontaneously. Then in 2018 I began collaborating with my mental health department, always through the association. […] I decided I wanted to do it, so I went first to my psychiatrist and told her: “I want to do this.”

In F.'s account, the PSW role does not emerge as a rupture from her past but rather as a natural continuation of relational practices already established before the training. Her narrative highlights that PSW identity does not originate in the training course; rather, the course serves to acknowledge and formalise an identity already in practice. The spontaneous work carried out in the tailoring workshop constitutes the concrete starting point of her peer support trajectory. In this way, F. positions herself as a subject already integrated within services, possessing a recognised capacity for agency that enables her to affirm and legitimise her role autonomously and proactively.

### 3.2 Training

The training phase revealed divergent positionings among participants: S. maintained a critical stance towards services, rejecting institutionalisation; M. embraced the training as transformative, emphasising relational learning; and F. focused on strategic self‐disclosure. Despite these differences, all three used the training to refine their understanding of the PSW role and its boundaries.

#### 3.2.1 S.'s Positioning: …Free From Roles Defined by Illness and Care

**I:** ‘Which class did you like the most?’

**S:** ‘The one about advocacy, rights, and intercession, because I'm part of an association called “Diritti alla Follia” [“Rights to Madness”], and there it's really about the rights of people with psychosocial distress—rights that are not respected—and I feel this issue very strongly … maybe because I'm a Libra, justice, I don't know…’

In S.'s account, subjectivity is constructed in explicit rupture with the institutional logics of services, which tend to keep people within predefined boundaries—formally included, yet without genuine emancipation. Her identity as a PSW arises from a claim to critical autonomy and independence, taking on an explicitly political connotation. S. overturns the rhetoric of the ‘*grateful patient’*, positioning herself instead as a witness capable of critically reinterpreting services on the basis of her own lived experience, refusing to be trapped within their logic.

This positioning is also expressed through her refusal to identify with a merely decorative role inside the service. S. rejects the idea of becoming an institutionalised figure—an innocuous, reassuring symbol who, while formally recognised, lacks real transformative power. She places herself explicitly outside services, assuming an independent and critical stance.

I almost felt like vomiting when talking about community mental health services, because I lived my experience in psychiatry so badly … it's not like afterwards I see the services as my saviors, you know!?

This narrative condenses the core of her positioning: not as a ‘saved’ subject, but as a critical figure who has traversed the system with pain, emerging with an autonomous vision.

S. also radically critiques the stepwise institutional pathway, which offers an illusory progression but in fact perpetuates the identity of ‘*service user’.* In this logic, the PSW role risks becoming merely the final step within the institution, functional to maintenance rather than to liberation:

It's almost like they give you a consolation prize, right!? “Do you want to become a PSW?” […] First hospitalization, then community, then supported housing, and finally you become a PSW. […] What I always criticize is that it feels like the goal is to keep people there, not to help them get out […] you also have to accept that you may never get out of that situation definitively.

Here, S. denounces a form of *domesticated recovery*, where well‐being is equated with adaptation rather than with truly overcoming the condition of being a patient. Instead, she envisions a path of definitive detachment from institutional identity, breaking with the paradigm of functional chronicity and imagining a subjectivity that is fully autonomous, free from roles defined by illness and care.

#### 3.2.2 M.'s Positioning: …Training Is in Relationships

In the Ex‐In course there was really a change, I started to fly because the course gave me this enormous awareness and so, afterwards, everything is wonderful, you fly. For me, the falcon is a marvelous animal; so I feel like I'm flying like a falcon, high up in the sky among the clouds, looking at the world from above and being able to see things as they really are.

The metaphor of the falcon represents a powerful symbolic construction of subjectivity. M. describes the training course as a device that enabled a new positioning: a lucid and detached gaze, capable of seeing ‘*things as they really are’*. This transformation marks emancipation from the role of service user, experienced as an elevation that is both subjective and epistemic. To fly means to see differently and from another perspective: the change concerns not only the person but also the way of knowing and inhabiting the world.

In groups of people who have experienced this kind of distress, receiving understanding and freedom of expression and exchange is enriching […] The training of “relationships” is that of the group, what happens in the group is enriching to the fullest.

For M., the peer group becomes an alternative space of subjectivation. That is where true training occurs: through reciprocal recognition, freedom of expression and the co‐construction of relational knowledge. For her, experiential training takes shape more within the group dynamic than through the frontal delivery of content.

In retrospect, M. also attributes to the training an ethical‐relational learning: the value of waiting, of difference and of subjective timing. This element consolidates her positioning as a PSW capable of welcoming, supporting and understanding others not only through acquired knowledge but also through the sharing of a relational stance.

#### 3.2.3 F.'s Positioning: …Choosing What and How to Share About Oneself

**I:** I imagine that during the course you shared your stories, and I wanted to ask if you experienced any difficulty in doing so?

**F:** In sharing it, no; in choosing what to share, yes, a little—because the recovery story is something deeply intimate, and there are things you may have only ever spoken about with the practitioners you most trust. Talking about those things I had never brought up in front of so many people—even though I trusted them—was a choice, about what and how. […] The backlash, when you talk about certain things, is there, it stays. But you deal with it. It was very useful, it was extremely liberating.

For F., narrating her recovery story is central to the training process but also a delicate act requiring attention, selection and awareness. The challenge lies not in speaking itself but in deciding what to disclose and how, calibrating depth of sharing to context and trust. This positioning reveals a subjectivity attentive to emotional balance, able to care for herself even while exposed.

Her agency emerges in the strategic management of self‐narration: she not only recounts her experience but directs it, intentionally choosing which aspects to highlight. Thus, her positioning as a PSW is not just that of a witness but of a reflective narrator, exercising control to protect integrity and turning exposure into an act of strength and self‐determination.

### 3.3 PSWs in Action

As PSWs began their roles, their positionings further evolved and took shape: S. asserted her experiential knowledge as crucial, challenging traditional clinical approaches; M. navigated the tension between empathy and professional boundaries; and F. embraced a fluid identity, integrating service user and professional roles. All three grappled with institutional dynamics, each finding unique ways to assert their value within mental health services.

#### 3.3.1 S.'s Positioning: …Now I Trust Myself and I Know Better Than Anyone Else What I Truly Want

What I have lived through cannot be explained to me by a practitioner—that is what experiential knowledge means. Only I can tell you what I went through. The mistake they make (referring to practitioners), in my view, is that the first thing they focus on is getting you to take medication. But do you know what it means for me to be here? That's what they should ask: “what happened to you?” You see!? Instead, they don't ask you what happened. (From the practitioners' point of view) you have a symptom, you have a severe depression, so they say: “you have to take medication.”

For S., experiential knowledge is not secondary or inferior but an embodied truth that cannot be delegated or replaced by technical expertise. Her positioning challenges psychiatry's epistemic centrality, contesting the symptom–medication logic and instead claiming an approach grounded in understanding the subjective meaning of suffering. She denounces the erasure of her story when diagnosis substitutes individual narration, with clinical intervention risking the interruption of meaning through prescriptive explanation.

S.'s positioning is rooted in a profound epistemic agency: the claim to know herself and navigate the world from an autonomous, coherent standpoint. This orientation also emerges through faith—not only spiritual, but as radical trust in her own discernment.

I am no longer conditioned by what the priest says, or the neighbor, or the dog, or the cat, or the canary, or the pharmacist, or the doctor, or the psychiatrist…. I have come to understand what I want and what I don't want—but above all, I know what is good for me. And if I believe in something, I will achieve it if I feel it has to be that way. Even my faith journey, and my beliefs, guide me a lot: I am a person of faith, and when I make a choice, I entrust myself deeply, if I feel it is the right one for me.

For S., advocacy represents an ongoing commitment, pursued both in relationships with professionals and with the broader public, through training and awareness‐raising initiatives. In all these contexts, the positioning as an ‘independent PSW’ is constructed not only through the determination to challenge established clinical practices considered immutable within services but also through others' recognition of their disruptive contribution. This posture emerges strongly in the interview excerpts.

For me, the figure of the PSW is an independent PSW. Sometimes I'm also a bit of a pain in the ass, I mean, right!? I have a way of doing things that sometimes I go in pretty hard, huh … but I like doing it because I want to break through! Sometimes this thing is even appreciated because you say ‘well, we need people who tell you things a bit, without beating around the bush, right?’

Simultaneously, the positioning as an ‘independent PSW’ is constructed through constant monitoring of her own role, to perform it in a manner that is meaningful to herself. This involves a relational balance and emotional stability, developed reflexively both through internal dialogue and, more importantly, through exchanges with peers.

I mean, it can happen that I say, ‘I am the PSW’, and maybe take initiatives that I shouldn't take, you know … it's always a question of balance, of not losing sight of your role. However, it's useful to have someone to talk to when you find yourself in a complex situation—a way to discuss things, even among peers. It's useful to be able to talk about it, to be able to say, ‘This happened to me’.

For S., the meaning of her role as a peer is constructed in the realm of empathy, where she presents herself as a source of inspiration for others. The absence of this dimension produces a sense of distance from her role. Furthermore, her action extends to an ethical dimension, oriented towards advocacy practices. It is in this interweaving that her professional positioning takes full shape.

If I accompany someone but have a negative and pessimistic attitude, I wouldn't be very effective. Being a source of inspiration is important to me. I like the word ‘inspire’. And it's just as important to help the people directly involved become aware of their rights.

#### 3.3.2 M.'s Positioning: The Professionalism of the PSW Between Empathy and Distance, a Bridge Between Patient and Service

In her account, M. addresses with clarity a central issue for the PSW role: how to act in moments of crisis and, above all, how to negotiate one's positioning when tensions arise between the needs of the service user and the expectations of the service. Her reflection begins with the theme of limits, introduced independently as an element of protection for both herself and others:

I try not to get too involved, meaning I set boundaries. I support a young autistic man who sometimes calls me even at 10 p.m. […] But I've managed to maintain this distance, which isn't easy. […] The course taught me that you need boundaries and that I have to protect myself […] If I go into crisis, I also hurt myself. […] So yes, I'll answer you even at 10 p.m., but I don't let myself get too involved. I listen to you, I tell you what I think. I try to be professional, and then I can still go to bed.

The discourse on ‘*boundaries’* becomes the pivot through which M. constructs her professional subjectivity: limits are not seen as obstacles but as tools that sustain the role. The agency of the PSW here emerges as an awareness of one's own limits, balancing empathy with distance. The reference to ‘*professionalism’* signals her intention to position herself within a regulated field, where support does not equate to unlimited availability but requires a reflective and sustainable posture.

A second narrative juncture reveals the ethical tension between loyalty to the service user and the mandate of the institution. M. recounts an episode during her internship, when she was involved in persuading a young woman under house arrest to begin long‐acting injectable medication, against the initial will of the person and her family:

The service used me to convince a girl under house arrest to do the depot. […] I managed to change her mind. She's doing it now. But I realized that she has the right to choose.

Her retrospective awareness that ‘*a PSW is not the intermediary of the service’* represents a key moment in her identity reflection. She reinterprets the episode as a slide towards a hetero‐directed positioning—an imposed role in which her actions ultimately served institutional needs more than those of the individual she was supporting. She follows this experience with a firm stance:

My role is to stand by the user. After that, it's up to the doctor to convince her, not the PSW. […] The PSW has to do things for the user, enter into empathy with them.

Her account outlines a subjectivity forged in tension between two poles: institutional pressure to act as a ‘*bridge’* between patient and service, and adherence to a relational ethic grounded in alliance with the user. The narrative highlights a conflict between imposed and claimed positioning that permeates PSWs' everyday practices and shapes their professional self.

M.'s trajectory shows how training functions not only as skill transmission but as a reflective space where lived experience can be re‐elaborated and transformed. In this sense, the course becomes a device enabling her to redefine practice around an ethic of listening, responsibility and boundary setting.

This is a transformation that is continuously evolving. For M., allowing herself to be permeated by others' stories, as a catalyst for redefining her own, remains a space to be safeguarded. The boundaries necessary to sustain her role—as previously brought up—are transcended because her professional identity is also built on the value of maintaining contact between her own story and those of others.

Every day I take home a gratification that keeps me going. The users know who I am and what I've been through, they ask me how I managed to become who I am and they seek me out to be listened to, even if just for a chat. It's the exchange of experiences that makes my work not only a help for others, but a continuous growth for myself.

#### 3.3.3 F.'s Positioning: …Beyond the Boundaries of the Role

A central theme in F.'s account is institutional recognition. Her experience takes place within a service characterised by a cultural climate favourable to the integration of PSWs, where the head of department was already engaged in projects aimed at valuing experiential knowledge. This supportive context allowed for a clear positioning from the outset:

My psychiatrist told me: “When you are in the office with me for an appointment, that is one thing. As soon as you step outside the door, you are the PSW. So when we do activities, you are my facilitator, not my patient.”

However, formal recognition did not coincide with the immediate assumption of the role. F. describes an initial phase of observation, choosing to remain on the margins to understand team dynamics and avoid interfering with ongoing projects. This reflects her awareness of the delicacy of entering a professional context regulated by implicit balances and entrenched hierarchies. Entry into the team is depicted not as a rupture but as a gradual negotiation and adaptation, supported by key figures such as the nurse—whom she calls ‘*amazing’*—valued despite, or even because of, her frequent marginalisation within services. This attentive gaze towards professional inequalities reveals a subjectivity able to reflect on power relations within the workplace.

A significant aspect of F.'s positioning is her ability to sustain roles that seem contradictory: being a service user and, at the same time, a PSW in the same service. For her, the value lies not in abandoning the user identity but in articulating it fluidly alongside the professional one. Identity is presented not as a linear passage from one state to another, but as a dynamic coexistence of positionings:

The recognition of professionalism should not be influenced by whether or not you live with mental distress. Even if I know that the PSW's professionalism arises from one's own lived experience of suffering, engaging in helping relationships is a choice that goes beyond being a service user.

This stance introduces an implicit critique of the medicalised vision of recovery, according to which ‘*being well’* coincides with definitively leaving behind the role of user. F. instead proposes a more complex conception of recovery, one that includes the possibility of continuing a care pathway while simultaneously exercising a professional role.

Simultaneously, F. emphasises the importance of distinguishing her professional identity from that of a service user in discursive spaces where such a distinction ensures others' recognition of her professionalism. Indeed, identity positionings are modulated according to the interlocutors, the contexts in which they take shape, and the discursive actions pursued. F. highlights how, in the relationship with the Department of Mental Health, the fluid articulation between the identity of a service user and that of a PSW can be perceived as professional ambiguity. In this relational context, maintaining these two identity dimensions separate represents for F. an essential positioning, as it is a necessary condition for professional recognition and legitimisation.

In the department, I have been actively supported by the service as a PSW, I am involved in various training projects, and they have asked me to be an honorary and founding member of SIRP Piemonte‐Valle d'Aosta, precisely in my role as an expert by experience and not as a service user, recognizing my role and my training.

The recognition obtained by F. within the Department of Mental Health does not guarantee that the same occurs in other contexts. In the associative context, the second area in which she operates, F. recounts how the training and role of PSWs are constantly devalued, generating in her a strong sense of frustration:

In the association, instead, which I experienced much more than the service when I was unwell, I still feel the presence of a strong distrust towards PSWs. Here, PSW training is seen as an accessory and not the foundation of active and conscious participation of people, a point on which I deeply disagree and which often puts me in a position of criticism regarding the association's stance.

It is interesting to note how F. interprets this devaluation by distinguishing between the individual and structural levels. The tensions do not stem so much from the lack of formal recognition of the role, but from the association's inability to grasp its transformative potential in redefining the organisational and relational logics of services in a participatory and recovery‐oriented manner. F.'s critical position thus appears not as a mere request for professional legitimisation, but as a denunciation of a systemic limitation: the absence of a perspective capable of recognising the PSW as an agent of change able to challenge the relationships of subordination still present in care contexts.

A direct manifestation of this lack of recognition is the absence of remuneration (except for paid internships), which F. identifies as the main obstacle to the full exercise of her professionalism.

Certainly, the lack of economic recognition, of the professional profile and skills of a PSW at a structural and legislative level is an obstacle. Lacking this, the main obstacle for me is not being able to work as a PSW and being formally recognized as such. The willingness of the service to move in a recovery‐oriented direction based on psychosocial rehabilitation practices and the recognition they give to my training as a PSW is a great help to me. (…) Support among PSWs is for me the basis for maintaining a clear professional identity: comparing notes with my colleagues and, secondly, with other professions such as researchers, mental health workers etc. helps me to make the best use of the EX‐IN training tools.

The lack of economic and institutional recognition is described not only as a material limitation, but as a discursive signal of a still unstable identity placement within the service system. The effectiveness of the PSW role appears to be linked to the sharing of a common theoretical and value framework between peers and professionals: recognition is a bidirectional process, in which the PSW can fully act only in contexts that share their principles, while the service can orient itself towards truly participatory practices only if it recognises the PSW as a promoter of new perspectives on care.

F. emphasises the importance of the collective dimension: constant peer exchange becomes a space for identity co‐construction, where mutual recognition strengthens both individual professional identity and the collective legitimisation of the role.

Finally, her account addresses a particularly relevant tension: the fragility of PSWs and the institutional perception of their risk of crisis. F. challenges the narrative that frames PSW instability as a specific threat, while the crises of ‘trained’ practitioners are systematically normalised:

We all experience crises, it's often said, but for mental health professionals it seems that only when a PSW goes into crisis can harm be done, and this is often used as an excuse not to accept the role. […] The weight of a professional in crisis—who nevertheless remains a trained practitioner—is hugely underestimated, but no one ever talks about it.

With these words, F. claims not only the legitimacy of her role but also the urgency of a cultural shift in services, where crisis is not considered the exclusive domain of service users or PSWs, but a human component of care work. In this reflection, PSW subjectivity is no longer conceived as fragile or deficient, but as a site of ethical, epistemological and relational insight.

### 3.4 Results—Summary

Although different, the positionings of each participant are articulated along three common dimensions.

The first concerns the connection with concrete biographical turning points: professional identity does not emerge suddenly, but through recognisable passages that participants anchor to a tangible object or context—a letter, a classroom or a workbench—that translates biography into role and memory into competence. These elements, easily identifiable, provide useful insights for training: mapping such turning points from the outset, associating each with a concrete product (testimony, exercise and assignment), and assessing their use during internship or fieldwork, thereby valorising existing resources and enhancing the professional applicability of experiential knowledge.

The second dimension is the ability to occupy liminal spaces: being ‘*on the threshold’*—between collaboration and advocacy, personal identity and professional role, belonging to the team and maintaining critical distance—emerges as a specific competence, not a sign of ambiguity. PSWs move in and out of institutional frameworks, recognising both constraints and opportunities, sustaining a reflexive stance, and acting as a hinge between different worlds.

The third dimension is the ability to inhabit contradictions: working in a field marked by structural tensions—between user‐centred care and service mandates, psychiatrisation and recovery, institutional logics and transformative perspectives, and experiential and professional knowledge—without resolving them but by working through them. The positionings observed appear as situated practices of mediation, adaptation or reformulation, preserving the specificity of lived experience while collaborating with services.

Each participant assumed different positionings within these three dimensions, revealing plural and situated interpretations of the role, oscillating between institutional assimilation and autonomous claims, with a strong identity investment in constructing a reflective form of experiential knowledge.

S. navigates between independence from services and strategic engagement, transforming her experience into advocacy. By maintaining critical distance from institutional logics, she highlights the contradictions between psychiatrisation and recovery.

M. builds her professional identity in balancing transformative participation with institutional logics, confronting the contradictions of the PSW role to safeguard the centrality of the user's experience and promote changes in services.

F. emphasises the coexistence of dual identities—that of PSW and service user—demonstrating fluidity between complementary roles and agency in engaging with the tensions between experiential and professional knowledge.

These positionings are presented in detail in Table 5.

**Table 5:** In‐depth commentary on the main narrative positionings that emerged.

*Common dimensions emerging from the analysis* → *Positionings ↓*	Lived experience as a biographical turning point in the development of PSW competences	‘Inhabiting the threshold’ (stance towards the service, ethical tensions and agency)	‘Inhabiting contradictions’ (conflicts between institutional logics and personal perspectives)
Positioning 1: ‘Between independence and strategic engagement’ [S.]	In S.'s account, her long experience as both service user and family member converges in the writing—slow and carefully crafted—of the application letter for the EX‐IN course: 3 months of work, conversations with services and repeated revisions. That document, although originally conceived as a formal requirement, becomes the first act through which 25 years of experience are transformed into a symbolic credential of suitability for the role of advocacy defender. After admission, S. continues to refer back to that letter to articulate her vision, to demonstrate coherence between biography and the ethics of the role, and ultimately as the concrete basis of her actions in defence of users' rights. (*Excerpt 1—pre‐training*)	S. constructs a discursive positioning that defines her subjectivity as ‘independent’ from services, oriented towards rights advocacy and activism, without, however, assuming an attitude of total opposition. Her agency takes shape in an identity positioning that constructs distance from the system as a reflexive and resistance practice, an act of critical vigilance that counters the risk of institutional subjectivation or reduction to a ‘token figure’ within the apparatus. (Excerpt 3*—*PSW in action)	Her narrative also highlights the contradictions between the logic of psychiatrisation and recovery perspectives: while the service claims to adopt a recovery‐oriented approach, in her view, it tends in practice to favour processes of chronification of subjectivities within the psychiatric context. S. positions herself critically in relation to these dynamics of institutional control; her positioning thus manifests a reflexive and strategic agency, capable of challenging the logics of control and claiming spaces of autonomy for herself and for service users. (Excerpt 2*—*Training)
Positioning 2: ‘Between the role of “peer” and that of “professional”’ [M.]	For M., the key turning point is her ‘*return to school’* after a long interruption in her studies. At first, this step generated uncertainty—‘*I didn't feel up to it’*—but overcoming the entrance interview and participating actively in the group enabled her to transform a previous educational fragility into a resource. M. shares this experience with the young people she works with, presenting her trajectory as an example of a concrete new beginning and thus turning a biographical limitation into a motivational lever that strengthens her function as a peer supporter. (*Excerpt 1—Pre‐training*)	M.'s story shows how the professional identity of the PSW is constructed within a constant tension between transformative participation and institutional normalisation. Her experience reflects a boundary narrative positioning, traversed by ethical and relational dilemmas. M. neither rejects the institutional logic nor accepts it passively: her account illustrates the possibility of inhabiting an interstitial professional position, in which the PSW role is continuously negotiated between institutional mandate and fidelity to the subjective experience of both herself and the users. The ethical tension emerging in her words—particularly concerning the therapeutic relationship and the management of conflicts between user and service—reveals that the role of PSWs also lies in their capacity to inhabit contradiction, transforming it into an opportunity for professional reflexivity and service practice change. (*Excerpts 2—Training; Excerpts 3—PSW in action*)	M.'s account highlights the contradictions inherent in the PSW role, particularly those between adherence to and attempts to reformulate service logics, as well as between a user‐centred approach and the orientation towards the institutional mandate. M. makes explicit the ethical tension that permeates her professional practice. Called upon to support the requests of the service, she realised that she had initially reproduced dynamics she had intended to challenge. Her narrative thus illustrates a process of awareness and re‐positioning, aimed at asserting the centrality of the user's choice and safeguarding their self‐determination. (*Excerpt 3—PSW in action*)
Positioning 3: ‘Between ex‐user and PSW’ [F.]	For F., the turning point is the internship in a tailoring workshop carried out before the EX‐IN training. While helping a person with tremors cut fabric, she experienced—without yet recognising it—the first dynamics of facilitation. Later, as a PSW, she reinterprets that episode as an ‘*in nuce’* experience: she recalls it in team meetings to illustrate the kind of practical support she can offer, different yet complementary to that provided by traditional professionals. (*Excerpt 1—Pre‐training*)	F. offers an additional perspective that challenges the dichotomous simplification between institutionalisation and transformation. Her narrative identity is constructed through the explicit valorisation of the coexistence of two seemingly opposing roles: that of a service user and that of a PSW. F. does not interpret her recovery as a definitive renunciation of the user role; on the contrary, she emphasises the richness of being able to sustain both positionings in different spaces and times, rejecting the traditional equation whereby ‘*being well’* would mean abandoning the user position. Her account highlights an agency that lies in the ability to move fluidly across different identities, no longer viewed as irreconcilable but as constitutive elements of a professional identity that is fluid, relational and deeply situated in context. (*Excerpt 3—PSW in action*)	Her account also highlights the tensions encountered by the PSW, who is called to confront the asymmetry between experiential and professional knowledge—often perceived within services as being in conflict. (*Excerpt 3—PSW in action*)

## 4 Discussion

The three positionings that emerged (Table 5)—though not exhaustive—represent distinct ways of inhabiting the role of PSW within the service participation. The findings show that there is no single way of being a PSW, but rather a constellation of dynamic configurations negotiated between institutional mandates, personal vision and relationships experienced within training and professional contexts. This plurality constitutes an epistemic and ethical resource, which should be safeguarded. Training, when understood as a reflective and relational space, can sustain this process, fostering the emergence of complex and non‐normative subjectivities capable of inhabiting thresholds and opening new meanings in relationships with others.

The narratives illustrate positioning as a fluid narrative space, traversed by transitions, movements and intermediate postures—distant from rigid dichotomies (such as co‐optation vs. resistance)—and populated instead by hybrid forms of belonging and agency, between normativity and autonomy, recognition and critique, and participation and distance. In line with the perspective that conceives PSW identity as processual and relational, constantly negotiated between subjective experience and organisational expectations [6, 7], this study analysed the narrative positionings of three participants to highlight the concrete ways in which PSWs inhabit their role within institutional practices.

These findings confirm and extend what has been observed in the literature regarding the tension between institutionalisation and the radicality of experiential knowledge [11, 14, 17]. In line with Repper and Carter [3], practices of agency emerge that resist normalisation, but unlike studies describing a progressive assimilation, our data document a plurality of positionings even within institutional contexts. This is in line with the findings of the study by Simpson et al. [31], according to which the emerging identity of PSWs is a liminal identity, in which PSWs found themselves ‘betwixt and between’ the multiple roles they performed. The narrative analysis underscores that PSW identity does not evolve in a linear fashion but through negotiations and, at times, active forms of resistance [20]. Comparisons with international research [26] suggest that, also in Italy, training may function as a critical space, partially removed from domesticated recovery [25] and capable of reworking experience beyond dominant codes.

The decision to adopt positioning analysis proved strategic in investigating subjectivity as a situated narrative construction, overcoming static representations and capturing the plurality of forms taken by experiential knowledge. We observed how participants navigated across different positionings in relation to professionalism, collaboration with services, the use of their personal stories, and relationships with others. The theoretical framework further allowed exploration of agency not only as explicit assertion but also as narrative choice, silence, posture or modulation of one's presence within services—reflective practices often invisible within standard evaluation models.

In our study, the involvement of the three PSWs took place through participant validation practices, conceived not merely as a technical check of transcripts but as a dialogical and reflective moment within the research process. This approach goes beyond the instrumental restitution of interviews for accuracy checking [40], framing validation as a co‐construction of knowledge aimed at accurately representing participants, correcting potential misunderstandings and making the interpretive pathway transparent [41].

Following Buchbinder [42], we shared a preliminary analysis with participants, offering them the opportunity to confirm, reformulate or contest our interpretations, thereby stimulating a collective reflection on the meaning of the narrated experiences. This step enabled us to critically interrogate our role as researchers and the semantics of power [43] that shape the analytic process, while recognising the epistemic value of participants as co‐producers of knowledge [44].

As noted by Madill and Sullivan [45], involving participants in the interpretative process not only enhances the trustworthiness of results but also fosters critical reflection on one's own positions, promoting a transformative understanding for both researchers and participants through a reflexive dialogue on the phenomenon under study.

## 5 Conclusion

This study has highlighted that there is no single way of being a PSW, but rather a constellation of possible positionings intertwined with each individual's biographical trajectory and lived experience. The narratives collected show that PSW identity is not a fixed or prescribable category, but an open and dynamic configuration, constantly negotiated between institutional mandates, personal understandings of the role, and relationships within training and professional contexts.

This plurality should be recognised as an epistemic and ethical resource for service users. The plurality of ways in which the PSW role can be interpreted represents a resource for users, as it broadens the possibilities for connection with diverse recovery experiences. Emphasising plurality does not mean reifying new ‘types’ of PSWs; rather, it acknowledges that each individual trajectory is unique and, precisely for this reason, can expand the range of proposals, relational postures and forms of support available—making them more responsive to the diversity of users' experiences and needs.

From this perspective, safeguarding plurality becomes an objective in its own right—not an obstacle to integration, but a necessary condition for developing more flexible, participatory and less standardised services that are responsive to the needs and preferences of each individual. Training, when conceived not merely as the transmission of skills but as a reflective and relational space—encompassing inner dialogue and reflective self‐analysis—can support this process by fostering the emergence of complex, non‐normative subjectivities and by helping each PSW to ‘find their place’ not within a functional grid, but within the living fabric of relationships and care practices.

For PSWs themselves, continuous dialogue with the diverse voices of colleagues is essential to maintaining a reflective stance and, in cases of direct collaboration with services, to preventing the risk of being assimilated into the dominant service culture. Supporting alternative ways of ‘being a PSW’ helps to avoid the crystallisation of service logics, practices and dynamics. This plurality, therefore, constitutes a fundamental condition for promoting more inclusive services, capable of providing differentiated and person‐centred forms of support.

It is precisely in this intermediate space—between individual biography and collective mandate, between lived knowledge and institutional context—that the transformative potential of the PSW role unfolds. This potential does not lie in conformity, but in the capacity to inhabit thresholds, to position oneself with awareness and to open up new meanings within relationships.

### 5.1 Clinical Implications

The plurality of ways in which the role of PSWs is understood and enacted should be regarded as a resource rather than a source of ambiguity or lack of clarity, and therefore actively safeguarded. This perspective is particularly relevant in the ongoing debate on the standardisation of training pathways, which highlights a tension between two needs: on the one hand, the definition of shared training content to ensure the credibility and accreditation of the role; on the other hand, avoiding standardisation that may compromise the authenticity and flexibility of peer support.

Findings from our research suggest that targeted regulation ensuring the inclusion of core themes in training programmes—such as the concept of recovery and the advocacy of rights [46]—may be useful for maintaining the culture of peer support. However, it is essential that these themes are not translated into rigidly codified technical competencies. The risk is that transforming the principles of peer support into technical procedures could lead to a regulation of the PSW's ‘way of being’, thereby reducing the diversity of approaches through which individuals construct their PSW identity. At the same time, diversifying training models, rather than adhering to a single standard, may help to preserve this variety, in continuity with peers' and individuals' personal understanding of mental health.

In light of our research findings, a further clinical implication concerns the need to give voice to tensions and to ‘remain on the threshold’, that is, to inhabit the boundary space between proximity and difference that characterises PSWs' work. To support this stance, services should provide spaces for autonomous reflection and peer‐to‐peer discussion. Ensuring a space in which differences among PSWs are made explicit and legitimate may help reduce the risks of assimilation while simultaneously preserving opportunities for action and transformative intervention. However, as one participant pointed out, the possibility of giving voice to these tensions would be greatly strengthened by the existence of an autonomous peer movement.

Training, and particularly peer supervision, should not assume a prescriptive function; rather, it should support processes of recomposing the polyphony of voices and tensions that traverse the experience of peer work. From this perspective, complexity is not an obstacle but a fundamental resource for keeping the meaning of the PSW role alive and generative.

### 5.2 Limitations

To our knowledge, this is the first systematic attempt to apply Positioning Theory to PSWs. In the absence of comparable studies, the three profiles should not be seen as exemplary or definitive, but as a starting point for further exploration. The self‐nomination of participants, all trained in the same EX‐IN programme and situated in a specific organisational context, limits the generalisability of findings. The positionings analysed emerge from small stories, strongly situated in the narrative scene and interaction, and do not provide a comprehensive account of PSW practices or identity trajectories.

Although adopting a precise methodological framework offers refined analytical tools, it also carries the risk of over‐interpretation, which we mitigated through analytic transparency and participant validation. These results should therefore be read as exploratory hypotheses, useful for guiding future comparative studies that include different contexts and perspectives.

## Author Contributions

**Ludovica Aquili:** formal analysis, writing – original draft, methodology, data curation, conceptualisation. **Michele Rocelli:** conceptualisation, data curation, formal analysis, writing – original draft, methodology. **Susanna Brunelli:** validation, data curation, writing – review and editing. **Federica Mangione:** data curation, writing – review and editing, validation. **Milena Negri:** data curation, writing – review and editing, validation. **Giuseppe Salamina:** writing – review and editing, supervision, visualisation, funding acquisition. **Elena Faccio:** conceptualisation, data curation, formal analysis, writing – original draft, methodology, project administration, supervision.

## Ethics Statement

Approval for the study was obtained from the Ethical Committee of the School of Padova, at the University of Padova (approval no. 4862).

## Consent

All patients provided written informed consent before enrolment in the study. The participants were informed of their right to withdraw at any time. They signed a written informed consent form regarding their participation in the research and its publication.

## Conflicts of Interest

The authors declare no conflicts of interest.

## Permission to Reproduce Material From Other Sources

Does not fit.

## Data Availability

The data that support the findings of this study are available on request from the corresponding author. The data are not publicly available due to privacy or ethical restrictions.
